# Establishing *Babesia bovis*-Free Tick Colony Following Treatment of the Host with Diminazene Aceturate (Berenil)

**DOI:** 10.3390/pathogens10050554

**Published:** 2021-05-03

**Authors:** Sharon Tirosh-Levy, Asael Roth, Binyamin Leibovich, Ludmila Fleiderovitz, Ohad Frid, Daniel Yasur-Landau, Ricardo Wolkomirskyi, Monica L. Mazuz

**Affiliations:** 1 Division of Parasitology, Kimron Veterinary Institute, Beit Dagan 50200, Israel; AsaelR@moag.gov.il (A.R.); borisl@moag.gov.il (B.L.); Ludaf@moag.gov.il (L.F.); ohadfrid@yahoo.com (O.F.); daniely@moag.gov.il (D.Y.-L.); Ricardow@moag.gov.il (R.W.); monical@moag.gov.il (M.L.M.); 2 Koret School of Veterinary Medicine, The Robert H. Smith Faculty of Agriculture, Food and Environment, The Hebrew University of Jerusalem, Rehovot 7610001, Israel

**Keywords:** *Babesia bovis*, *Rhipicephalus (Boophilus) annulatus*, babesiosis, bovine, diminazene aceturate

## Abstract

*Babesia bovis* is a widely-spread tick-borne hemoparasite of cattle with major economic and animal welfare consequences. *Rhipicephalus (Boophilus) annulatus* is a one-host tick which transmits bovine babesiosis in the Middle East and Africa. Laboratory rearing of ixodid ticks is essential for the investigation on ticks or tick-borne diseases. Establishing a tick colony in the laboratory usually originates from ticks harvested in the field, which may be naturally infected with various pathogens. This especially applies to carriage of *B. bovis* as it is highly prevalent in endemic areas and is transmitted transovarially in ticks. Here, we describe the use of diminazene aceturate (Berenil) in order to establish laboratory colonies of *Babesia*-free *R. annulatus*, from ticks collected in the field. Ticks collected in the field were kept until oviposition and hatched larvae were introduced to naïve calves, which led to infection of the calves with *B. bovis*. Calves were then treated with diminazene aceturate several times until the engorged ticks dropped. The eggs and larvae collected from these ticks were parasite-free, as demonstrated both by infection of splenectomized calves and by PCR. This suggested protocol is a useful tool to create parasite-free tick colony and may, theoretically, also be beneficial to reduce parasite circulation in the field, although not recommended, as resistance to diamenizene aceturate might develop.

## 1. Introduction

Bovine babesiosis is a tick-borne disease of cattle caused by protozoan parasites of the genus *Babesia*, order Piroplasmida, phylum Apicomplexa. The disease is widely distributed and has major economic and animal welfare consequences in the cattle industry. The main species of *Babesia* infecting cattle in Asia, Africa, Australia, Central and South America are *B. bovis* and *B. bigemina,* which are transmitted by ticks of the *Rhipicephalus* genus [1,2,3,4]. 

As *Babesia* parasites are bloodborne parasites which infect the host red blood cells, clinical signs are mainly the result of intravascular hemolysis and characterized by fever and hemolytic anemia. *Babesia bovis* is considered more pathogenic and infections may sometimes manifest in neurological signs as a result of sequestration of infected erythrocytes in cerebral capillaries [3,4]. Recovered animals may remain subclinical carriers for prolonged periods of time and can be a source of infection for ticks. *Babesia* parasites are also transmitted transovarially in ticks and, therefore, both cattle and ticks are involved in the maintenance of the parasites in endemic areas [3,5,6].

Clinically affected animals can be cleared from parasites following treatment with antiparasitic drugs, mainly diminazene aceturate or imidocarb. However, eradication of the disease in a specific area requires elimination of the vector ticks. In endemic areas, prevention of clinical disease usually involves a combination of immunization, chemotherapy in acute cases, and tick control practices [3].

As ticks are important animal parasites and are vectors of various veterinary and human pathogens, studies investigating the transmission, infectivity, and pathogenicity of tick-borne diseases often use tick bites as the natural mode of infection under laboratory conditions. These studies require the maintenance of laboratory colonies of hard tick vectors [7]. Tick colony in the laboratory usually originates from ticks harvested in the field, from either animals or the environment. When ticks are collected in endemic areas, they may be naturally infected with various pathogens. This especially applies to carriage of *Babesia* parasites, due to their high prevalence in endemic areas worldwide [2], and to their transovarian transmission in ticks.

Israel is endemic for both *B. bovis* and *B. bigemina*, with *Rhipicephalus (Boophilus) annulatus* being the major vector for these parasites [8]. Here, we describe the use of diminazene aceturate (Berenil) in order to establish laboratory colonies of *Babesia*-free *R. annulatus*, from *B. bovis* infected ticks collected from animals in the field. 

## 2. Results

### 2.1. Tick Colony #1

Ticks were brought from the field and engorged ticks were kept until oviposition. Larvae hatching from 0.5 g of eggs were placed on a calf (#883) that had been treated with diminazene aceturate one and three days before the introduction of the ticks. An additional treatment was administered three days after the ticks were placed. The calf was monitored daily and displayed no clinical signs of babesiosis. However, on day 17, few *B. bovis* parasites (>0.01% parasitized erythrocytes) were observed in the calf’s blood-smear. Therefore, additional treatments of diminazene aceturate were administered on days 17, 18, and 20. On days 21−24, engorged ticks dropped from the calf, and were kept until oviposition and hatching (Table 1).

A sample of the eggs laid by ticks collected from calf #883 was tested by PCR and found negative for both *B. bovis* and *B. bigemina*. Larvae from 0.3 g of the hatched eggs (second generation) were placed on a second calf (#901). The calf was monitored daily and did not display clinical signs and parasites were not detected in its blood-smears. The calf was treated with diminazene aceturate on days 19 and 21, before the ticks started to drop. On days 21−24, engorged ticks dropped on their own and were kept until oviposition and hatching (Table 1). 

Larvae from 0.3 g of the hatched eggs (third generation) were placed on a splenectomized calf (#903). This calf was relatively young (two months old) and presented a basic higher temperature above 39 °C. Between ten and 16 days after the introduction of ticks, an intermittent increased body temperature, up to 40.1 °C, was noted on three days. The packed cell volume (PCV) remained stable (32−34%) and no parasites were detected in its blood-smear (Table 1). The ticks that dropped from this calf were considered free of *B. bovis.* A sample of eggs from the ticks that were collected from calf #903 was PCR-negative to both *B. bovis* and *B. bigemina*.

### 2.2. Tick Colony #2

Ticks were brought from the field and engorged ticks were kept until oviposition and hatching of larvae. Since, in this collection, only few engorged ticks were collected, all hatched larvae were placed on a calf (#183). On day 8 after introduction of the ticks, *B. bovis* parasites were observed in the calf’s blood-smear (Figure S1), and on day 10, its PCV dropped from 30−33% to 25%. Blood collected from the calf on day 8 was PCR-positive for *B. bovis* and PCR-negative for *B. bigemina* (Figure S2). The calf was treated with diminazene aceturate on days 8, 10, 15, 17, and 20. On days 21−24, ticks dropped from the calf, and were kept until oviposition and hatching (Table 2).

The blood collected from calf #183 on day 8 (12 mL) was injected to another calf (#181). On days 12−15 after injection, *B. bovis* parasites were observed in blood-smears and the calf had fever (39.9−40.2 °C) and anemia (PCV dropped from 28−30% to 17−19%). On day 16, the infection resolved without treatment, no parasites were observed in the blood-smear and the calf’s body temperature and PCV stabilized. On day 30, this calf was splenectomized, and on day 33, small numbers of parasites were detected in its blood-smear, with no clinical signs. Thereafter, intermitted low-level parasitemia was observed in its blood-smear for at least three months (Table 2). 

A sample of the eggs from the ticks that were collected from calf #183 was PCR-negative to both *B. bovis* and *B. bigemina* (Figure S2). Larvae from 0.3 g of the hatched eggs (second generation) were placed on a splenectomized calf (#187). The calf was monitored daily and did not display clinical signs, and parasites were not detected in its blood-smears. The ticks that dropped from this calf were considered free of *B. bovis* (Table 2). 

## 3. Discussion

Investigating vector-borne diseases under semi-natural conditions in the laboratory is important to gain applicable knowledge of parasite–vector–host interactions, transmission, and pathogenesis. In order for such scientific investigation to be robust, infection of ticks with specific pathogens should be controlled, and the ticks used should be generally specific pathogen free (SPF). Infection of ticks with pathogens may also affect ticks’ survival and fecundity, as has been demonstrated for *B. bovis* in *R. annulatus* [9]. Rearing ticks in the lab requires maintaining favorable environmental and nutritional requirements, which may differ between ticks species [7]. This also applies to the one-host tick *R. annulatus* [10,11]. 

Establishing a tick colony in the laboratory involves collecting a tick seed stock from the field. In endemic areas, these ticks are likely to be infected with *Babesia* parasites [2]. When infected larvae are fed on a naïve calf, clinical signs of babesiosis usually develop after about 10 days for *B. bovis*, and about 14 days for *B. bigrmina* (since the infecting life stages are larva and nymph, respectively) [3]. In both cases presented here, ticks were infected only with *B. bovis* and the calves developed clinical signs 8−10 days after the introduction of the larvae. Infection of calf #181 also demonstrated how infection is usually contained by healthy animals without treatment, and both clinical signs and detectable parasitemia in blood-smear spontaneously disappear after four days. However, when the calf was later splenectomized, parasitemia re-appeared, revealing that the calf was not cleared of parasites without medical treatment. 

In order to clear the ticks from infection, we treated their host with diminazene aceturate. The two treatment protocols described here were designed to achieve different aims when collecting ticks in the field. In the first trial (colony #1), the only purpose was to establish a pathogen-free colony; therefore, the calf (#883) was treated before the introduction of the ticks to lower or prevent infection. This regime did not prove efficient for prevention, however, it delayed the onset of parasitemia (which occurred 17 days after tick infection), and led to very low parasitemia. Further treatments, while the ticks remained on the calf, cleared both the ticks and the host. The second trial (colony #2) was designed not only to establish a pathogen-free colony, but also to obtain *B. bovis* isolate from the field. To do so, the treatment of the calf (#183) was withheld until initiation of clinical signs. This allowed to collect parasitized blood from the calf before treatment, which cleared both the ticks and the host. 

Since *B. bovis* is transmitted transovarially in ticks, any infection that was brought from the field would be transferred between generations and maintained in the tick colony. Therefore, in order to demonstrate clearance from infection, it was necessary to examine the next generation of ticks that hatched in the laboratory. Here, we used both conventional (introducing the ticks to splenectomized calves) and molecular techniques (PCR) to ensure that the ticks were parasite-free.

Although calf #903 had elevated body temperature ten days after the introduction of ticks, it was probably unrelated to *B. bovis* infection, as the ticks were negative by PCR and no parasitemia was detected in its blood-smear. The cause of the increase in body temperature could not be explained by the laboratory and clinical examination, and may be related to its young age (2 months) and poor adaptation to ambient heat load. This trial was performed in the summer, where the temperature in Israel could be very high— up to 40 °C. It has been shown that body temperature of young calves increases with increase of ambient temperature and body temperature was higher in hot weather [12]. 

Currently, managing babesiosis in endemic areas involves treatment of only clinically affected animals in combination with environmental prophylaxis and the use of acaricides [3]. Moreover, early exposure or vaccination of calves against *B. bovis* is recommended in endemic areas to develop immunity against clinical disease, with strategic tick-control [13,14]. Theoretically, the fact that treatment of infected calves can lead to clearance of parasites in ticks may suggest that prophylactic chemotherapy in highly endemic areas may be used to reduce *Babesia* yield in ticks. However, the protocol presented here included repeated doses of diamenizane aceteruate, which normally does not occur in field, and should not be recommended, since the development of diaminazene aceturate resistance can occur [15]. Moreover, the effect of the same protocol on *B. bigemina* infected ticks was not evaluated in this work, as collected ticks were not infected with this parasite, and should be further investigated in areas were both parasites are present. These implications should be further investigated in order to re-define management and control practices in endemic areas.

## 4. Materials and Methods

### 4.1. Tick Collection and Laboratory Keeping

Ticks were collected manually from infected cattle from grazing beef cattle herds from the area of the Golan Heights, Israel. Engorged female *R. annulatus* ticks were washed in double distilled water (DDW) and placed in 9-mm Petri dishes (up to 25 ticks per plate). The Petri dishes were incubated at 28 °C and 85% relative humidity. Egg laying usually started after three days and lasted approximately two weeks. After oviposition, eggs were monitored every 3 days, until changes in eggs color were observed and approximately one third of the batch was collected. Eggs from several ticks were weighed together and 0.3−0.5 g of eggs were incubated at 28 °C and 85% relative humidity for 19 to 21 days, until hatching. Unfed larvae were kept at 28 °C and 85% relative humidity, until the end of the pre-feeding period, and were placed on a calf 10 to 14 days after hatching.

### 4.2. Calves’ Maintenance, Tick Introduction, and Monitoring

Four-week old calves were purchased from a commercially intensive, zero grazing dairy farm. The farm was free of babesiosis and calves were not exposed to ticks. Calves were kept in individual stalls surrounded by a water barrier to avoid dispersal of ticks. Unfed larvae were placed on the dorsum of each calf. After introduction of the larvae, calves were monitored daily for clinical signs, including measurements of body temperature and PCV, and inspection for parasites in blood-smear. After 21−30 days from the introduction of the larvae, engorged adult ticks dropped from the calf and were collected from the floor of the stall. When the majority of ticks had dropped, calves were sprayed with amitraz 12.5% (Taktic, Intervet International BV, The Netherlands) every 2−3 days for 3−5 times, to ensure the calves were no longer infested.

### 4.3. Splenectomy Procedure

Calves were isolated and fasted for 24 h prior to the procedure. An area on the left flank of approximately 30 cm^2^ was closely clipped and prepared aseptically. Anesthetic protocol included xylazine (0.2 mg/kg, intramuscularly) and ketamine (2 mg/kg, intravenously). The animal was placed in right lateral recumbency and secured to the operating table with cotton ropes. Perioperative fluid therapy was initiated by administration of Ringer’s lactate solution (3−5 mL/kg/hr) during the procedure. Local anesthesia of the incision site was achieved by injecting lidocaine HCl 2% subcutaneously. An incision of approximately 15-cm long was made parallel and about 4 cm posterior to the last rib. The spleen was then manually withdrawn, and double ligatures (No. 3 chromic catgut) were applied to the splenic blood vessels 2 to 3 cm from the hilus, which were cut distal to the ligatures to extract the spleen. The peritoneum and musculature were sutured with No. 3 chromic catgut. Intraperitoneal antimicrobial treatment (Pen-Strep 1 mL per 25 kg—Norbrook) was administered prior to closure. The skin was sutured with No. 2 supramid. After the procedure, animals were monitored during recovery and were ambulatory within one hour of surgery, and were treated with NSAIDs (flunixin meglumine—2.2 mg/kg, intravenously) for three days.

### 4.4. Diminazene Aceturate Treatment 

Calves that were treated with diminazene aceturate (Berenil) received doses of 3.5 mg/kg, injected intramuscularly. 

### 4.5. DNA Extraction and Polymerase Chain Reaction (PCR)

DNA was extracted from bovine blood and from tick-eggs using the DNeasy Blood and Tissue Kit (Qiagen, Hilden, Germany) according to the manufacturer’s instructions. For DNA isolation from tick eggs, eggs were stored at −80 °C until use and 100 mg of eggs were macerated with a glass rod prior to DNA extraction, as previously described [8]. Parasite DNA was detected by nested PCR specific for *Babesia bovis* and *Babesia bigemina*, as previously described [16]. 

## 5. Conclusions

In this study, we proposed two protocols to achieve and maintain *B. bovis*-free *R. annulatus* tick colony in the laboratory. One aiming to establish a pathogen-free tick colony, with minimal clinical effect on the calves used as hosts, and the other aiming both to isolate the parasite from the ticks brought from the field and later to clear the tick colony from infection. We demonstrated that treating tick-infected calves with diamenizene aceturate clears both the calves and the attached ticks and that it also prevents transovarian transmission ticks, with future generations remaining free of parasites. This protocol proved useful in the laboratory and may, theoretically, also be beneficial to reduce parasite circulation in the field.

## Figures and Tables

**Table 1:** The study design and outcome of establishing *B. bovis*-free tick colony (colony #1) from infected ticks collected from the field.

Calf	Day	Treatment/Clinical Signs
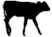	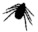	***R. annulatus* from field cattle** → 0.5 g eggs → larvae
**#883**	−3	Diminazene aceturate
	−1	Diminazene aceturate
	0	Larvae deposition on calf #883
	3	Diminazene aceturate
	17	*B. bovis* detected in blood-smear, Diminazene aceturate
	18	Diminazene aceturate
	20	Diminazene aceturate
	21−24	Engorged females drop-off
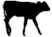	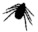	***R. annulatus* from #883** → 0.3 g eggs → larvae
**#901**	0	Larvae deposition on calf #901
	8−18	No CS
	19	Diminazene aceturate
	21	Diminazene aceturate
	22−24	Engorged females drop-off
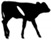	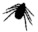	***R. annulatus* from #901** → 0.3 g eggs → larvae
**#903**	0	Larvae deposition on calf #903, splenectomized
	10	Fever 40.1, no parasitemia
		No CS

CS—clinical signs.

**Table 2:** The study design and outcome of establishing *B. bovis*-free tick colony (colony #2) from infected ticks collected from the field.

Calf	Day	Treatment/Clinical Signs
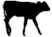	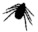	***R. annulatus* from field cattle** → eggs → larvae
**#183**	0	Larvae deposition on calf #183
	8	Fever, anemia, *B. bovis* detected in blood-smear, blood collection
	8	Diminazene aceturate
	10	Diminazene aceturate
	15	Diminazene aceturate
	17	Diminazene aceturate
	20	Diminazene aceturate
	21−24	Engorged females drop-off
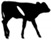	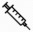	**Infection with 12 mL infected blood from #183**
**#181**	0	Larvae deposition on calf #181
	12−15	Fever, anemia, *B. bovis* detected in blood-smear
	16	Recovered
	30	Splenectomy
	33-	*B. bovis* in blood-smear intermittently
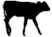	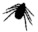	***R. annulatus* from #183** → 0.3 g eggs → larvae
**#187**		Eggs → Neg PCR
	0	Larvae deposition on calf #187, splenectomized
		No CS
	21−24	Engorged females drop-off

## Data Availability

Data is contained within the article.

## Supplementary Materials

The following are available online at https://www.mdpi.com/article/10.3390/pathogens10050554/s1, Figure S1: Low parasitemia, with singular *B. Bovis* parasites detected in blood-smear of calf #183, eight days after the introduction of *R. annulatus* larvae, Figure S2: Negative PCR results for the presence *B. bigemina* in the blood of calf #183, 8 days after the introduction of *R. annulatus* larvae, and of *B. bovis* and *B. bigemina* in eggs laid by *R. annulatus* ticks which dropped off calf #183. (PC-positive control, NC-negative control, M-DNA 1 KB ladder).
