# Supplementary figures and images for: Establishing Babesia bovis-Free Tick Colony Following Treatment of the Host with Diminazene Aceturate (Berenil)
Source: Pathogens. 2021 May 3;10(5):554. doi: 10.3390/pathogens10050554 (PMC8147767; doi:10.3390/pathogens10050554)

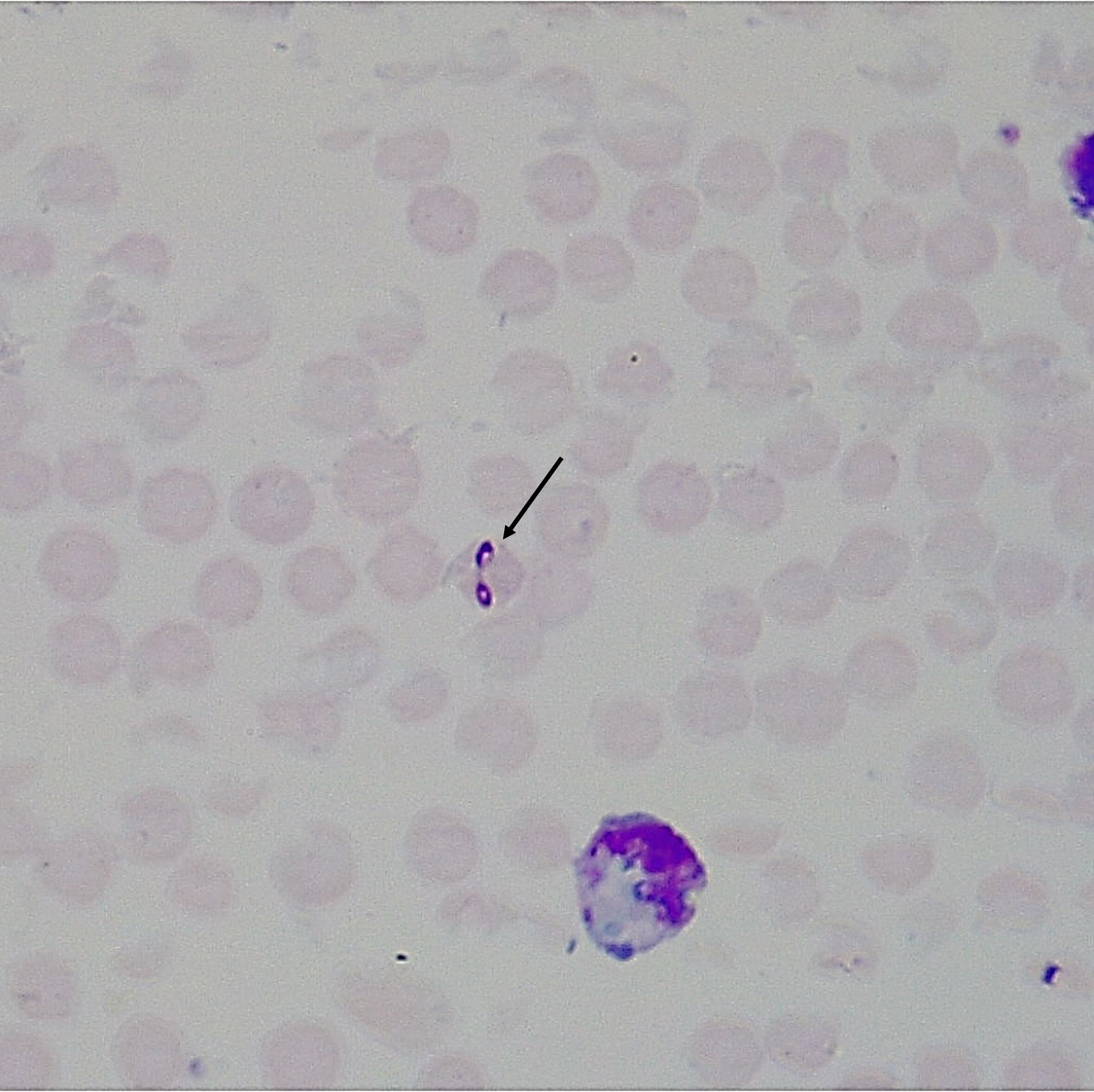

Supplement: Supplementary file 1 [file pathogens-10-00554-s001.zip › S1.png]

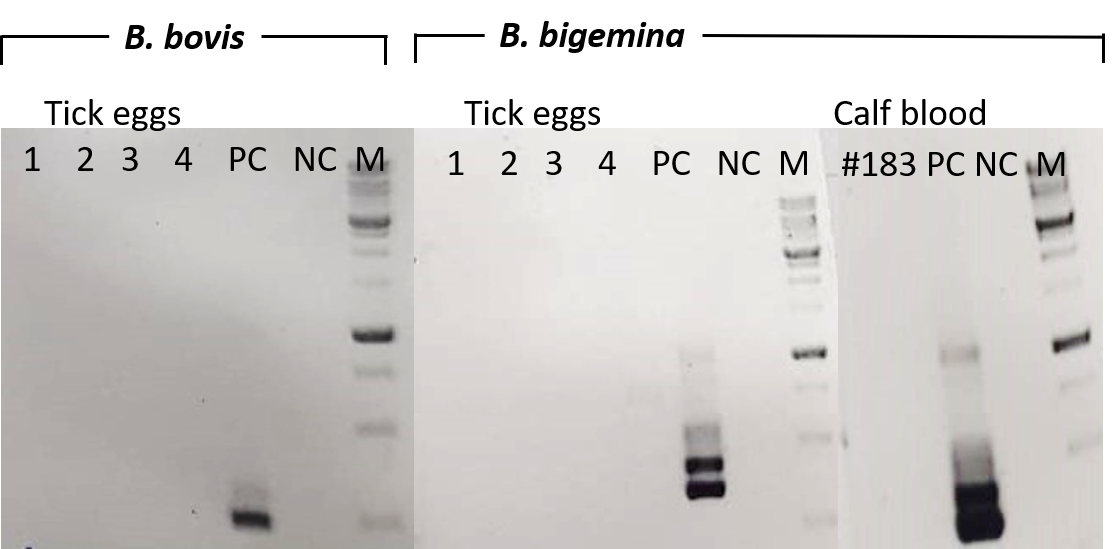

Supplement: Supplementary file 1 [file pathogens-10-00554-s001.zip › S2.png]
